# Radiofrequency catheter ablation as a treatment option in a patient with hypoplastic left heart syndrome and atrial flutter after Fontan operation—Case report

**DOI:** 10.1002/joa3.12579

**Published:** 2021-06-24

**Authors:** Marta Jagosz, Wiktoria Kowalska, Radosław Lenarczyk, Mateusz Knop, Roland Fiszer, Beata Średniawa, Ewa Jędrzejczyk‐Patej

**Affiliations:** ^1^ Student Scientific Society Department of Cardiology Division of Medical Sciences in Zabrze Medical University of Silesia Katowice Poland; ^2^ Department of Cardiology Congenital Heart Diseases and Electrotherapy Silesian Centre for Heart Diseases Zabrze Poland; ^3^ Division of Medical Sciences in Zabrze Department of Congenital Heart Diseases and Pediatric Cardiology Medical University of Silesia Katowice Poland; ^4^ Division of Medical Sciences in Zabrze, Medical University of Silesia, Katowice, Poland, Department of Cardiology, Silesian Center for Heart Diseases, Zabrze, Poland Katowice Poland

**Keywords:** atrial flutter, Fontan operation, hypoplastic left heart syndrome, radiofrequency catheter ablation

## Abstract

We report a 15‐year‐old male with hypoplastic left heart syndrome (HLHS) after Fontan operation with recurrent, drug‐resistant atrial tachycardia. With the use of electro‐anatomical mapping system (EnSite) an atrial flutter (AFl) with reentry activation around the tricuspid valve was diagnosed. Successful radiofrequency catheter ablation (RFCA) was performed.

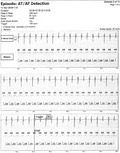

## 1 INTRODUCTION

Patients with complex heart diseases with a morphologically single ventricle require staged palliation which is usually the Fontan operation. Since its inception, the procedure has undergone a lot of modifications to improve hemodynamics, which resulted in longer life expectancy of these patients. Despite the improvement in surgical and interventional techniques, many patients after Fontan operation develop atrial tachycardia (AT) with increasing frequency of arrhythmia episodes during the follow‐up that poorly respond to pharmacological treatment. Although radiofrequency catheter ablation (RFCA) could be an optimal invasive treatment for supraventricular tachyarrhythmias, the limited access because of multiple surgical interventions in patients with complex congenital heart diseases may be a challenge.

We report a case of a child with lateral tunnel Fontan and an epicardial pacemaker who underwent RFCA for drug resistant atrial flutter (AFl).

## 2 CASE REPORT

A 15‐year‐old child with complex congenital heart disease, after multiple hospitalizations because of persistent, drug resistant supraventricular tachyarrhythmia was admitted to a high‐volume tertiary care university center presenting atrial tachycardia with ventricle response of about 130 bpm. He had been diagnosed as a newborn with a hypoplastic left heart syndrome, mitral stenosis, and aorta atresia, initially palliated with a modified Norwood operation in 2003, followed by Rashkind operation and balloon angioplasty of coarctation of the aorta 3 months later. Then he underwent hemi‐Fontan and Fontan operation followed by tricuspid valve and left pulmonary artery plasty. Because of a Fontan operation, he developed sick sinus syndrome which was treated by epicardiac dual chamber pacemaker implantation in September 2018.

**FIGURE 1:**
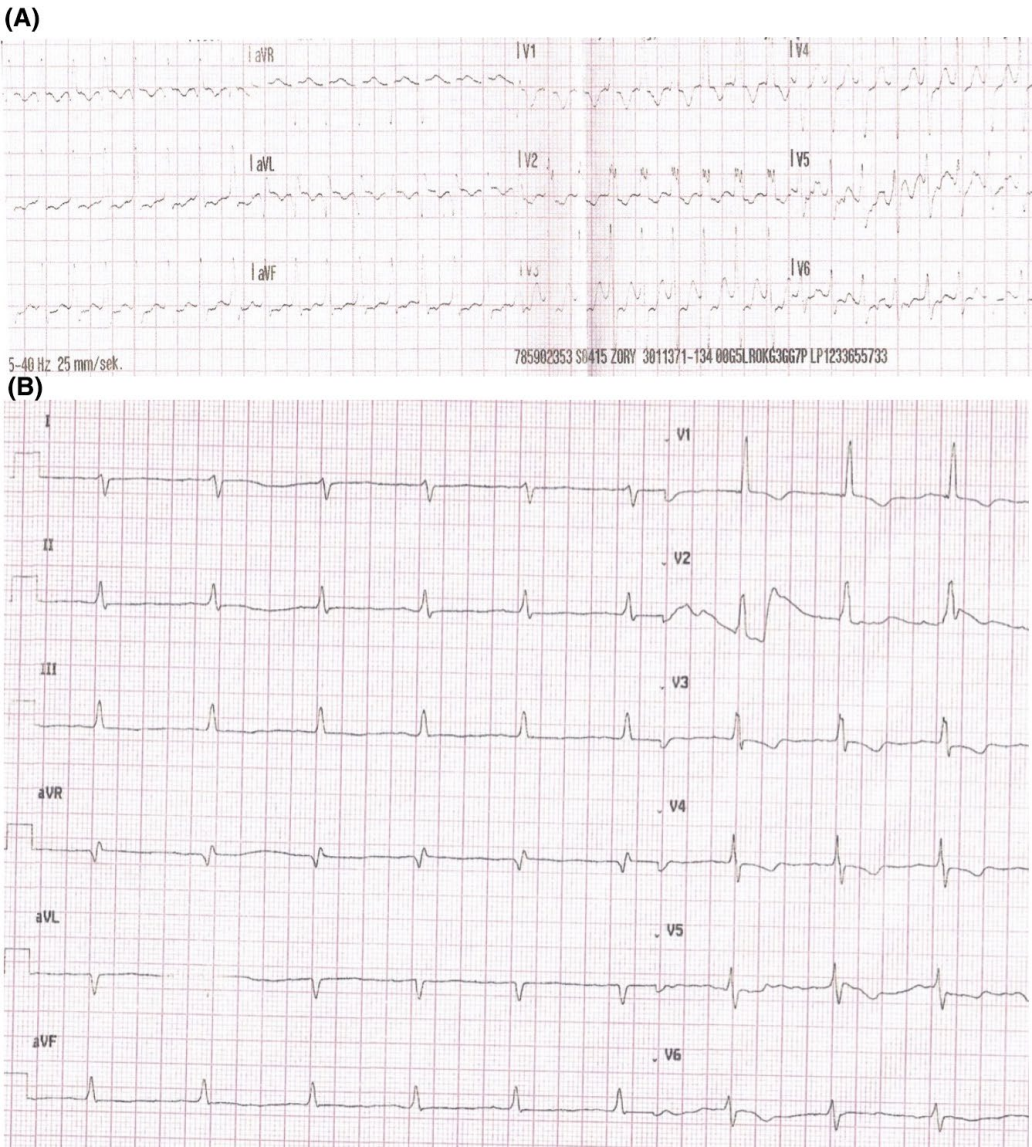
ECG strip (25 mm/s) demonstrating the arrhythmia (A) before the ablation and the sinus rhythm (B) after ablation

**FIGURE 2:**
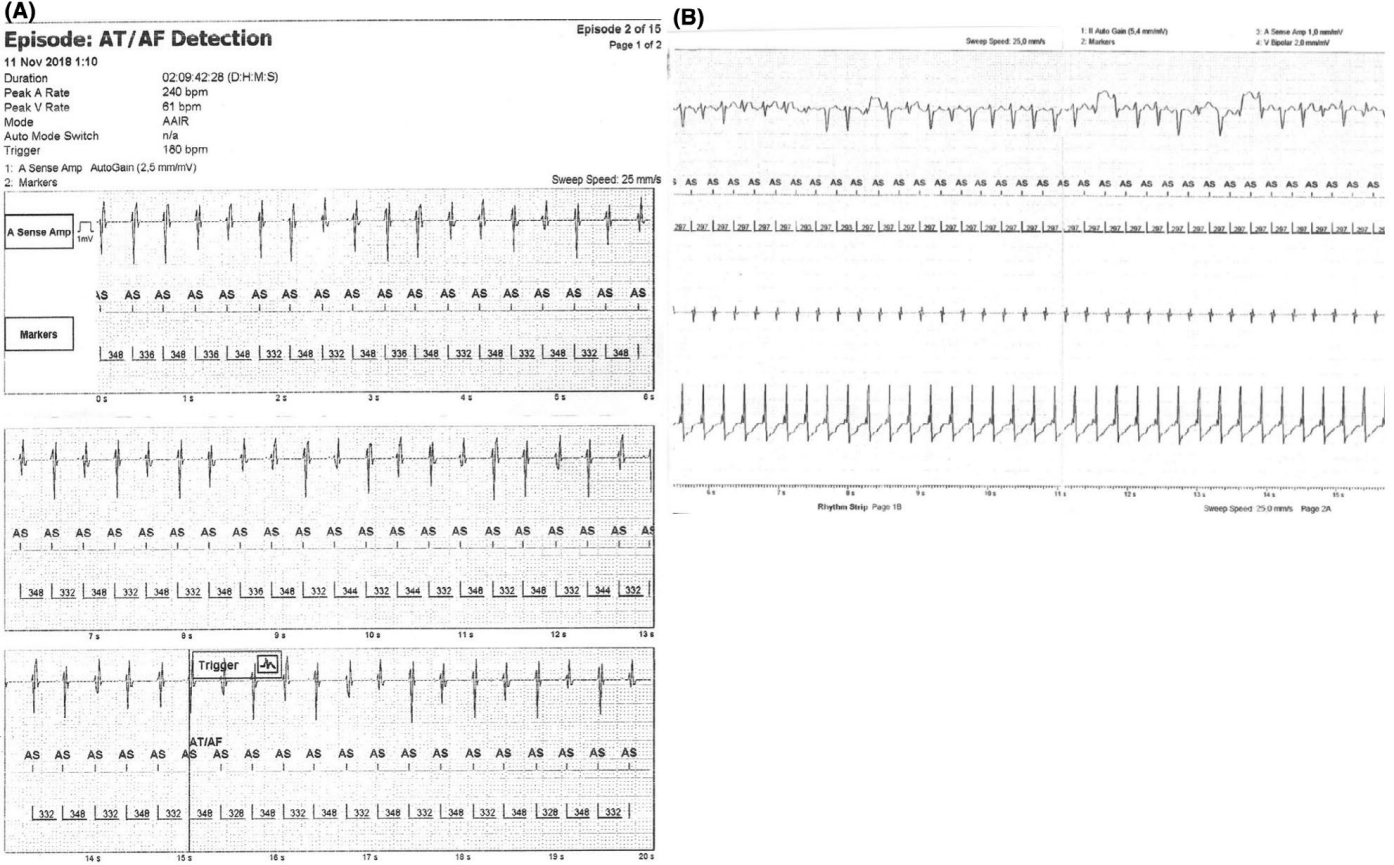
Intracardiac electrograms (IEGM) demonstrating the arrhythmia recorded by the pacemaker

During the admission he complained about heart palpitation and significant functional impairment. Physical examination showed a blood pressure 109/77 mm Hg and an O_2_ saturation of 93%. In the ECG the atrial tachycardia with a ventricular rate of 130 bpm was observed (Figure 1). Atrial flutter was also found in the routine pacemaker check‐up (Figure 2). Echocardiography demonstrated a single, morphologically right ventricle which was connected through a large ventricular septal defect with hypoplastic left ventricle. A good contractility of a single ventricle with ejection fraction of 54%, small regurgitation of a single atrio‐ventricular valve, and a presence of a single vessel above the single chamber with laminar blood outflow were observed. The fenestration with medium 5 mm Hg resting pressure (maximum 9 mm Hg) was visible and there was no fluid in pericardium or in pleura.

After 2 days of hospitalization and treatment with amiodarone the conversion to sinus rhythm was not obtained. Because of multiple, recurrent hospitalizations because of persistent, drug resistant supraventricular tachyarrhythmia the decision of a Heart Team was to qualify the patient to RFCA.

**FIGURE 3:**
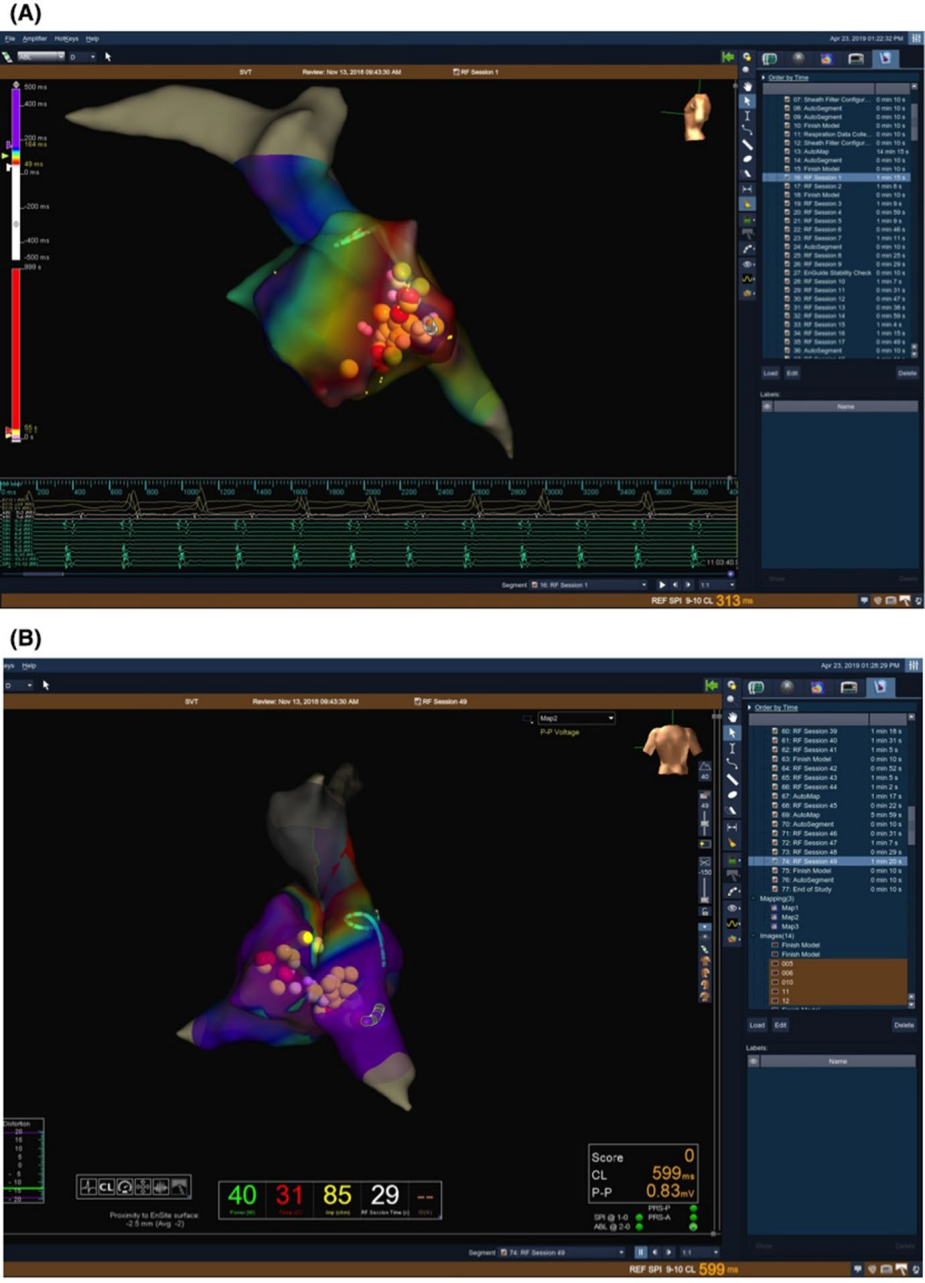
Right atrial activation (A) and voltage (B) map acquired using EnSite Cardiac Mapping System. Points of RF application are marked as dots

The procedure was performed in November 2018. Before the procedure transesophageal echocardiography was performed to exclude the thrombus in the heart. The ablation was performed in cath lab in general anesthesia. The dose of 2500 IU/L heparin was administered. The procedure was complicated because the vascular access was very much limited. Both femoral veins were in atresia. In addition, during the second Fontan procedure the inferior vena cava (IVC) was cut off and connected to the pulmonary artery—the new vascular canal was also connected with right atrium (RA) via fenestrations. Because of the atresia of femoral veins, a hydrophilic leader and a long vascular sheet were used to reach the vascular canal. With the use of Lasso catheter, the canal was reconstructed (on the EnSite Cardiac Mapping System) and atrial reference signals (from that vascular canal, at the level of the RA) were recorded. Subsequently, with the use of transseptal sheet, the RA (via fenestrations) was reached to create an activation and potential map of RA. A reentrant tachycardia (AFl with the cycle of 300 ms) around the tricuspid valve, with the isthmus between the tricuspid valve and the scar that was created by the excision of the SVC and creation of the vascular canal during the second Fontan procedure was found (Figure 3A and 3B). The application line in the isthmus, that is between the tricuspid valve and the scar was performed. As the anatomy was largely changed by multiple previous surgical interventions, and there was no IVC anymore, the ablation was performed in an “equivalent” of a cavo‐tricuspic isthmus (as for typical AFl ablation). During ablation, the AFl terminated, and was not induced any more by programmed stimulation. Because of the complicated anatomy of the congenital heart disease the confirmation of the bidirectional block in the isthmus was technically impossible to perform. The duration of the procedure (skin‐to‐skin) was 135 minutes. The radiation exposure was 62 mGy, 635.3 DAP. There were no complications after the procedure. In ECG and Holter ECG tests there was no AFl recurrence. Three days after the procedure the patient was discharged. The pharmacological treatment after the RFCA was warfarin according to INR (with the target level 2.0‐3.0), metoprolol (25 mg bid), and enalapril (1.25 mg bid).

During 20 months of follow‐up the patient was in general good condition, without any syncope and remained free of arrhythmia.

## 3 DISCUSSION

Atrial arrhythmias following Fontan‐type surgery are very common and increase in the postoperative time reaching about 50% of patients during 20 years of follow‐up and are associated with significant morbidity and mortality. Furthermore, almost half (47%) of these tachyarrhythmias are resistant to antiarrhythmic drugs.

Several isthmuses of tachycardia could be developed by anatomical sectors made by the orifices of vena cave, coronary sinus, suture lines of atriopulmonary anastomosis, or lateral tunnel repair. Catheter ablation in post‐Fontan patients is challenging because of distorted anatomy of the heart after multiple surgical interventions consequently barriers to access the native atrial tissue, limited access to the heart or possible hemodynamic instability. It is even more challenging in patients with lateral tunnel or extracardiac conduit because the caval veins are not directly connected to the heart and the access to the heart is possible only through the fenestration if present.

Furthermore, the acute and long‐term results of RFCA in Fontan patients are significantly worse than in other congenital heart diseases with a high risk of recurrent tachycardia, which reaches 40%. For these reasons, RFCA in these group of patients is not very common. There is a case report of a 7‐year‐old boy with HLHS after Fontan procedure who required RFCA because of drug resistant reentry atrial tachycardia. The ablation electrode could not have been inserted through small fenestration. After dilatation with the use of angioplasty balloon, the RFCA was successfully performed. Other case report of a 24‐year‐old female after Fontan operation has shown efficacy of RFCA for paroxysmal AF. However, pulmonary vein stenosis was developed as a consequence of the ablation procedure. Moore et al. observed that AT of extracardiac total cavopulmonary connection (E‐TCPC) in 36 patients was treated with success via catheter ablation in 83% of the cases.

## 4 CONCLUSION

Radiofrequency catheter ablation with the use of electro‐anatomical mapping system could be effective and feasible treatment of drug resistant atrial flutter in pediatric patients with hypoplastic left heart syndrome after Fontan operation.

## CONFLICT OF INTEREST

Ewa Jędrzejczyk‐Patej and Radosław Lenarczyk—consultant fees from Medtronic, Biotronik, Abbott, and Boston Scientific. Beata Średniawa—consultant: Medtronic, Zoll, Bayer, lectures fee for: Boehringer‐Ingelheim, Bayer, Pfizer.

